# The use of virtual slides in the EUROPALS examination

**DOI:** 10.1186/1746-1596-6-S1-S23

**Published:** 2011-03-30

**Authors:** Jan G van den Tweel, Fred T Bosman

**Affiliations:** 1 UMC Utrecht,Department of Pathology H4.312, Heidelberglaan 100, 3584 CX Utrecht, The Netherlands; 2 UMC Maastricht, Department of Pathology, P. Debyelaan 25, 6229 HX Maastricht, The Netherlands

## Abstract

**Background:**

The only realistic way to improve harmonisation of European pathology training is to define the generally accepted competencies and to test them periodically during the training programme (progress test). The European Association of Pathology Chairs and Program Directors therefore decided to implement an annual on-line test using virtual slides in addition to static jpeg images and theoretical MCQ’s. The EU supported this endeavour as EUROPALS (EUROpean Pathology Assessement & Learning System).

**Methods:**

To address the challenges of large scale digital testing EUROPALS teamed up with i-Path Diagnostics Ltd, a company specialising in utilisation of virtual slides in histology/pathology education and examination. Specific examination software was used in the test system.

**Results:**

In the first 2 years we provided at five occasions progress tests, including 2 proctored tests, attracting hundreds of participants. The accessibility varied from suboptimal to good and improved with each subsequent test. It was influenced both by the hosting server capacity and the internet bandwidth at the user’s location.

**Conclusion:**

On-line testing using virtual slides is possible but requires a good collaboration between the provider and the user. Both should be aware of the requirements and threads of large scale testing with hundreds of simultaneous users.

## Background

The European Association of Pathology Chairs and Residency Program Directors (EAPCP), founded in 2005 to meet the challenges of European (Pathology) integration, has come up with several new initiatives to meet the challenges in modern pathology. Although it seems logical to harmonise the different European training programs, the enormous differences between the national requirements makes this endeavour not realistic in the short term [1]. However, agreement on the competencies expected of a trainee at the end of his/her training is much easier to achieve. Therefore it was decided to develop well defined general and specific competencies for pathology trainees [2] and use this as a basis for an on-line European Progress Test for trainees and pathologists. The European Union supported this initiative in 2007 with an Erasmus grant (Erasmus LLL program EUROPALS) [3].

Delivering tests on-line requires sophisticated test management software. The test could be restricted to using standard jpeg images (static images), which is technically less challenging and follows a style similar to conventional paper-based evaluations. Virtual slides (whole slide images) are more demanding as they require the use of innovative image serving and viewing software and, because of the enormous size of the images, a storage facility capable of storing hundreds of gigabytes. However, they correspond much better to daily pathology practice [4].

Displaying the virtual slide, allows questions to be asked about the overall diagnosis without specifically highlighting the key diagnostic features (as one must in "field of view" photography). This mimics conventional glass slide-based examinations but without the need for a microscope [5] and in principle allows the deployment of the test all over Europe. Even if a question is asked on a specific field of view, the trainee can zoom out and see that field of view in overall context of the slide. This contextual information in virtual microscopy is essential in training pathologists, in both finding the key diagnostic fields within the complete slide or perceiving how a diagnostic field fits into the surrounding non-diagnostic tissue context. A key skill that a trainee pathologist needs to develop is to identify diagnostic areas within a complete slide. Restricting questions to defined fields of view on the slide limits the ability to assess this skill. If the aim of the on-line test is measuring diagnostic skill as opposed to theoretical knowledge, then virtual slides are an essential component. Configuring an on-line test in pathology requires flexibility over the question types that can be asked.

## Methods

### Hosting server architecture

Serving virtual slides as part of an on-line test is bandwidth and server intense, particularly if image latency is to be kept to a minimum. This is even more important when there are multiple users accessing the images simultaneously. A successful system requires multiple image servers, which are carefully load balanced. Load balanced, implies that when one server gets busy due to user activity another server takes over and provides the services that are being requested. For the EUROPALS test, multiple image servers operate in tandem allowing us to process multiple requests for images per second.

The number of synchronous participants (users) is also an important parameter for defining the required server capacity. The infrastructure at the server side must be sufficient to deal with more than the expected maximum number of users at any one point. The number of expected users should be pre-empted and the server architecture stress tested for the amount of simultaneous requests. Examination workflow may also put extra stress on the system as most users will progress through the exam at a similar speed and hence will all be accessing the same virtual slide file simultaneously. The number of users is an issue for the host to resolve and to ensure that the architecture is sufficient to supply the same information to a maximum amount of users at any given time during the exam. Often this is implemented by several caching strategies, where the server doesn’t need to read the whole image over and over again, but keeps (parts of) often requested images at an adequate resolution for serving over the web in its internal memory.

In the case of the EUROPALS examination this infrastructure is provided from a centralised hosted service in London that is connected to a main internet backbone that feeds the UK, Europe and the USA. Clearly the more image servers operating, the more efficient will be the service to any end client. However, the cost of providing larger infrastructure increases considerably. The benefit of using a hosted solution for online virtual slide exams lies in the management of the server infrastructure and the avoidance of capital expenditure by the exam administrator. The server infrastructure to deliver an online exam to multiple participants in multiple locations at a given time is substantial and much greater than that used to supply virtual microscopy on a daily basis to a few users at one site. Hence, the capital costs for deployed infrastructure and maintenance on an annual basis could be prohibitive to a test administrator, as the infrastructure would only be used during test periods. A very extensive and scalable test server facility as described above can provide superior infrastructure and can schedule several tests throughout the year for different test administrators to create greater efficiency for the infrastructure.

### Software management requirements

Configuring an on-line test in pathology requires flexibility over the question types that can be asked. EUROPALS is made possible by using the PathXL on-line test management platform. This allows easy configuration and editing of new MCQ and free text questions. Static images and virtual slides can be associated with each question; questions can be set on the entire virtual slide or on an annotated region of the slide and the complete test can be published on-line at the touch of a button.

An administrative system within the software allows new users to be defined with the automatic generation and emailing of passwords, allowing them to login and access the test. Each user has a unique identifier that allows information to be stored on their progress through the test and on their end results. In a large sample group it is essential to have this automated process to speed and ease the administration process prior to and after an exam.

With Examination Management Software, such as i-Path’s PathXL OLT, used in the EUROPALS examination the administrator can set many of the exam parameters, such as open dates and duration, in advance of the exam which enables participants to take the test at a convenient time and location for the participant. In PathXL OLT for the EUROPALS exam, the proctored Dutch and British tests were set to open only for the 3 hour duration of the test. The following Open Test for other European countries was set so that the test could be taken anytime within a two week window and once a participant logged in they had three hours to complete the test.

The statistical analysis and collection of results from a large participant group can be automated by Examination Management Software. Manual marking of papers would make such an ambitious project as EUROPALS extremely time consuming for the administrator. At the conclusion of a EUROPALS test the administrator may download results for either a single participant, group of participants or the whole cohort. For EUROPALS, PathXL OLT and i-Path automatically marked the multiple choice questions and compiled statistics on them.

### Server side issues

If the only goal of an on-line test is to poll and gather answers using textual presentation and MCQ selection or free text input, then this can be done with many off the shelf software solutions. However, the biggest challenge is one of incorporating high-resolution images and in particular virtual slides. Virtual slides are challenging in that they require a very dedicated software platform for delivering image content associated with text based descriptions and a question and answer panel. Within a EUROPALS test, virtual slides are presented in the same window as the question, making it easy to navigate the slide in its entirety, and subsequently answer the question set for that slide.

The PathXL platform can accommodate all of the major image formats including, but not limited to, Hamamatsu (.ndpi), Aperio (.svs), Zeiss (.mrxs), jpg and jpg2000. This provides additional flexibly in collecting examination material from several centres, where images may have been scanned on different instruments.

An average histopathology virtual slide can have a file size in the region of 1GB with 5 x 10 mm of tissue on the slide and scanned at x40 magnification. It is possible to have a much smaller size if there is less tissue on the slide. A standard jpg image will produce a file size in the region of kilobytes to a few megabytes [4]. The largest whole slide images are produced when scanning cytopathology slides, or slides requiring focus, as these glass slides are scanned in layers (Z-stack) to produce the required depth of focus [5]. The ideal number and space between layers is 21 layers and a spacing of 1.5 microns between layers [6]. With 21 layers and a spacing of 1.5 microns at x40 magnification a typical cytopathology glass slide will produce a 7.3GB virtual slide using i-Path’s image compression algorithms. The PathXL viewer is part of PathXL web browser and therefore unable to run as a stand alone viewer. It also runs through the standard internet port (80) which means that it will not be blocked by company firewalls.

### User side issues

For users, there are several technological considerations that may impact on their online exam experience using virtual slides.

User side issues mainly relate to the internet bandwidth at the user’s location. The speed of virtual slide delivery can be greatly affected by poor bandwidth at the user’s site. During the EUROPALS examination a virtual slide can be delivered via a bandwidth of a 1-2 MB/s. However better performance can be achieved with bandwidths of > 1GB/s where panning and zooming become very smooth and instantaneous.

A second priority for the user is to ensure there is sufficient RAM in their computer. A recommended level of RAM to ensure smooth navigation of virtual slides is 1GB. A lack of RAM in the PC may result in ‘jerky’ or a ‘tiled’ effect when navigating from one area of a slide to another.

Maximum virtual slide performance is achieved when a virtual slide is navigated in a style similar to a glass slide. During best virtual slide navigation practice, using low power then jumping to high power at one location, sends only one request to the image server. With poor practice, such as panning at high power around the slide and scrolling through all available magnifications, multiple requests for each tile and magnification requested are sent, thereby creating a longer ‘queue’ at the image server and subsequently a longer wait for the virtual slide to be delivered to each participant [5].

### Participants

Participants were recruited from all pathology training institutions around Europe, as well as from the group of practising pathologists. The first test attracted nearly 700 participants, the others around 300

## Results

Initially there was a problem with the accessibility of the system. The server capacity could not deal with nearly 700 simultaneous users. A second test 2 months later attracted 250 participants in a 2 week period. This test went without any problems. A third test was given for 40 British candidates simultaneously. No assessment problems were encountered. A fourth test for 100 Dutch candidates simultaneously showed access varying from slow to normal, probably as a result from low internet bandwidth at the user’s location. A fifth test for 200 persons some weeks later over a 2-week period went without problems.

## Discussion

The use of virtual slides in medical teaching, especially in practical settings, is becoming more and more common. Nevertheless we occasionally still encounter at our UMC occasional problems when students using 20 computers study the same virtual slide at the same moment (using another provider than iPath). Testing large numbers of students in test situations with many virtual slides is a challenging operation with many potential bottle necks. The EUROPALS test is as far as we know the first test that involves virtual slides that is simultaneously approached by hundreds of candidates from over more than 20 countries.

The test system as implemented in Europals has many advantages such as:

• Participants can access the test at the same time from locations all across Europe

• Entirely web-based system accessible from office, home etc

• All participants looking at the same section of the same block

• Test scores and immediate feedback and answers can be automatically provided at the end of each test

• Scores can be compared automatically against peer groups at the end of each test

• Virtual slides are supported from all major scanner manufacturers

• Questions can be created using standard jpeg images

• Questions can be created on annotated areas of a virtual slide

• Automatic user registration page

• Scores held centrally in database

• Secure login for all users

• Test can be configured to run at a specific date and time for a specific duration

• Single questions can be asked on multiple slides

• No need to manually score papers – automated system

However there are also problems that have to be solved:

• Unable to determine if individual users broadband speeds and computer processing power is sufficient to view virtual slides via the web

• The system is currently unable to determine if individual users have adequately sized monitors and screen resolution to view the virtual slides

• Image Serving such large image files grows more expensive as the number of simultaneous participants increases.

• Field of view smaller with a virtual slide in comparison to glass and therefore the amount of time required to “screen” a whole slide virtually will increase

• Users not familiar with navigating a virtual slide tend to zoom and pan, therefore queuing each tile being served by the image server and slowing the speed of delivery.

• Confirming the users identity – non-proctored users registering and taking the test with multiple email addresses

Dealing with large numbers of concurrent users across large geographical distances is the main challenge. The current i-Path server architecture supports the European Union and the USA comfortably. As the need for online tests based on virtual slides increases across the globe, both the server architecture of the host and the local bandwidth (and RAM) of the user must increase. On the host side a network of servers can be created across the globe to speed delivery of virtual slides, but this leads to duplication of slides on the different servers and increases the infrastructural requirements of delivering the test. The use of cloud computing might increase the performance and efficiency, since additional virtual image servers can dynamically be added to and removed from the infrastructure on demand. On the user side, there is considerable variation in bandwidth availability and standardization and computer hardware is impossible to control for. Hence, users with a high bandwidth and superior PC may have an advantage in that they will more easily be able to view the slide at high speed during the test. The issues around the speed of delivery have been addressed to some extent by i-Path on their PathXL platform by not serving whole images but rather serving the portion of the image that the user is currently examining.

Despite all the problems and growing pains, the system used is a promising tool for future tests. With all people involved working hard to solve the problems we are confident that virtual testing is going to play an essential role in future examinations.

## Competing interests

The authors declare that they have no competing interests.

## Ackowledgment

This study is supported by the EU project: **133852-LLP-1-2007-1-NL-ERASMUS-ENW**

This article has been published as part of *Diagnostic Pathology* Volume 6 Supplement 1, 2011: Proceedings of the 10th European Congress on Telepathology and 4th International Congress on Virtual Microscopy. The full contents of the supplement are available online at http://www.diagnosticpathology.org/supplements/6/S1

## References

[B1] Bosman FT van den Tweel JG Unison or cacophony: postgraduate training in Pathology in Europe Virchows Arch 2009 454 497 505 10.1007/s00428-009-0746-6 19255781

[B2] European Pathology Training Curriculum http://www.eapcp.org/Europals/training.htm

[B3] EUROPALS grant http://www.eapcp.org/Europals/europalsap.pdf

[B4] Huisman A Looijen A van den Brink SM van Diest PJ Creation of a fully digital pathology slide archive by high-volume tissue slide scanning Human Pathology 2010 41 751 7 10.1016/j.humpath.2009.08.026 20129646

[B5] Hamilton PW van Diest PJ Williams R Gallagher AG Do we see what we think we see? The complexities of morphological assessment Journal of Pathology 2009 218 285 291 10.1002/path.2527 19291709

[B6] Evered A Dudding N Accuracy and perceptions of virtual microscopy compared with glass slide microscopy in cervical cytology Cytopathology 2010 10.1111/j.1365-2303.2010.00758.x 20482714

